# Risk factors and early prediction of pancreatic cancer among patients with diabetes mellitus: a systematic review and meta-analysis

**DOI:** 10.3389/fendo.2025.1698850

**Published:** 2025-11-19

**Authors:** Haoru Cong, Jiamei Song, Le Liu, Shilin Liu, Haonan Wu, Zheng Nan

**Affiliations:** 1 College of Traditional Chinese Medicine, Changchun University of Chinese Medicine, Changchun, Jilin, China; 2 Department of Endocrinology and Metabolism, The First Affiliated Hospital to Changchun University of Chinese Medicine, Changchun, Jilin, China; 3 College of Pharmacy, Changchun University of Chinese Medicine, Changchun, Jilin, China

**Keywords:** diabetes mellitus, incidence rate, machine learning, pancreatic cancer, risk factors

## Abstract

**Aim:**

Diabetes mellitus (DM) increases the risk of pancreatic cancer (PC). This study evaluates risk factors for PC in DM patients and the predictive accuracy of machine learning (ML) models to provide research-backed data for the development and update of intelligent prediction tools.

**Methods:**

PubMed, Cochrane, Embase, and Web of Science were systematically retrieved, up to December 1, 2024. The quality of the original studies was assessed through the Newcastle-Ottawa Scale (NOS). A meta-analysis was conducted on the c-index that reflects the comprehensive accuracy of the prediction models.

**Results:**

18 studies were included. The rough annual incidence of PC among DM was estimated at 0.4% (95% CI: 0.1% - 0.9%), and the incidence rates of PC for new-onset DM and pre-existing DM were 0.3% (95% CI: 0.1% - 0.5%) and 0.5% (95% CI: 0% - 2.7%), respectively. The possible risk factors included age at DM diagnosis, weight changes, blood sugar, ALP, GI symptoms, pancreatic disease history, and the usage of hypoglycemic drugs. ML models based on risk factors had ROC-AUCs of 0.79 (95% CI: 0.75-0.84) in the training set and 0.79 (95% CI: 0.71-0.87) in the validation set.

**Conclusions:**

Risk factors for PC in DM are diverse. Current ML models appear to exhibit favorable predictive accuracy but are built on severely imbalanced data. Future studies with larger, broader populations are needed to address this limitation.

**Systematic review registration:**

https://www.crd.york.ac.uk/PROSPERO/, identifier CRD42025631534.

## 1 Background

Diabetes mellitus (DM) is a chronic metabolic disease caused by defects in the pancreatic islets and/or insulin function. As the disease progresses, the risk of organ failure will continuously increase. In 2021, the global number of DM patients reached 529 million, indicating a year-on-year increase trend. It is estimated that by 2050, over 1.31 billion people will suffer from DM (1). In addition, DM can induce severe macrovascular diseases (cardiovascular and cerebrovascular diseases) (2, 3) and microvascular complications (diabetic kidney disease, diabetic retinopathy) (4), peripheral vascular diseases (diabetic foot disease) (5), and malignant tumors (6), which become direct or indirect causes of death and disability. Therefore, the occurrence and progression of DM significantly affect the quality of life in patients, and gradually aggravate the social and economic burden.

Pancreatic cancer (PC), as a serious malignancy, has a short survival period. Multiple studies have demonstrated that DM is an independent risk contributor to the occurrence of PC (7). However, predicting the occurrence of PC in patients with DM remains clinically challenging. In order to effectively prevent PC, some researchers have explored the risk factors for PC in patients with DM. For instance, PC has been associated with the age of the diabetic patients, their smoking status, and their family history of digestive system cancers (8). Although these studies have described the risk factors for PC, there still seem to be some problems in specific prediction models based on risk factors in clinical practice. For instance, the lack of standardized and quantified tools makes the prediction results rather ambiguous, and the accuracy of the predictions is also questionable. Therefore, clarifying the risk factors for PC in DM patients and providing accurate predictive models for the incidence of PC in DM patients remain urgent issues to be addressed at this stage.

In recent years, machine learning (ML) has been widely used across medical research domains, because it can process high-dimensional data and predict the diagnosis, evolution and prognosis of diseases. Therefore, ML exhibits favorable value in clinical application. In the process of diagnosing and treating diabetes, the application of ML has received widespread attention. Some scholars applied ML to identifying the occurrence of diabetic retinopathy, demonstrating excellent clinical utility (9). Evangelos K Oikonomou et al. (10) evaluated the clinical value of ML in the prediction, diagnosis, and treatment of cardiovascular diseases associated with DM. ML provides a brand-new solution to the management of diabetic complications. In this context, some researchers applied ML to construct a prediction model for PC in DM patients, and conducted research on core clinical issues such as risk factors and rates of incidence of PC among diabetic patients.

However, up to now, there still exists an absence of systematic reviews to summarize those risk factors and the accuracy of models for predicting PC in patients with DM, which poses challenges to clinical work. As a consequence, this study was designed to systematically review the incidence rate of PC and its risk factors among patients with DM, and to evaluate the accuracy of predictive models, providing strong evidence for subsequent research in this domain.

## 2 Methods

### 2.1 Registration of the study

This research followed systematic review and meta-analysis reporting guidelines (**Additional Material 1**). It was prospectively registered in the PROSPERO platform (ID: CRD42025631534).

### 2.2 Eligibility criteria

Inclusion Criteria:

1. The subjects of this study were patients with DM.
2. A risk factor analysis was conducted, or a predictive model for the risk of PC was constructed.
3. Studies published in the English language.

Exclusion Criteria:

1. Unpublished conference abstract.
2. Studies do not strictly differentiate between PC and other tumors.

### 2.3 Data sources and retrieval strategy

A systematic retrieval of PubMed, Cochrane, Embase, and Web of Science up to December 1, 2024 was conducted, and subject headings plus free words were used, with no restrictions on region or period. The retrieval strategy is provided in **Additional Material 2**.

### 2.4 Literature screening and data extraction

The retrieved literature was loaded into Endnote. The titles or abstracts of the literature, after eliminating duplicates, were read to filter out the original studies that did not match. Then, after the downloading and reviewing of the full texts, the studies that met the inclusion criteria for this systematic review were ultimately selected.

Before data extraction, a piloted data extraction sheet was formulated following standardized guidelines, including the title, DOI, first author, publication year, first author’s country, study type, follow-up time, number of PC incidence cases, total number of PC cases, construction of the prediction model (yes/no), verification method of the prediction model, type of the prediction model, and risk factors.

The literature filtration and data extraction above were independently carried out by 2 investigators and cross-checked, and if there existed a disputed issue, a third investigator would assist in adjudicating.

### 2.5 Risk of bias in studies

The NOS scale (11) was adopted to evaluate the risk of bias in the original studies selected. The scale contained a large number of questions in 4 different fields. Among them, 2 points were awarded for comparability, and the rest of the questions were each worth 1 point. A total score of 7–9 points indicated a high-quality study; 4 to 6 points denoted a medium-quality study; and 0 to 3 points denoted a low-quality study. Two researchers independently conducted the bias risk assessment based on the NOS scale. Afterward, they cross-checked each other. If there existed any dispute, a third researcher would be invited to assist in adjudicating.

### 2.6 Synthesis methodology

A meta-analysis was conducted on the incidence of PC in DM. Because of the exceedingly low average annual incidence rate, the double arcsine transformation was employed during the analysis process. A meta-analysis was also performed on the indicators (c-index) used to evaluate the overall accuracy of ML models. In some of the original studies, when the 95% confidence interval (CI) and standard error of the c-index were missing, the standard error was estimated based on the study by Debray TP et al. (12). The heterogeneity index (I^2^) was used to assess the heterogeneity among the studies. The random-effects model was applied when I^2^ exceeded 50%, while the fixed effects model was utilized for I^2^ values of 50% or below. When conducting the meta-analysis of the c-index, we separately concluded the c-index of the training set and the validation set. The meta-analysis was performed in R 4.4.2, considering P-values <0.05 statistically significant.

## 3 Results

### 3.1 Study selection

**Figure 1:**
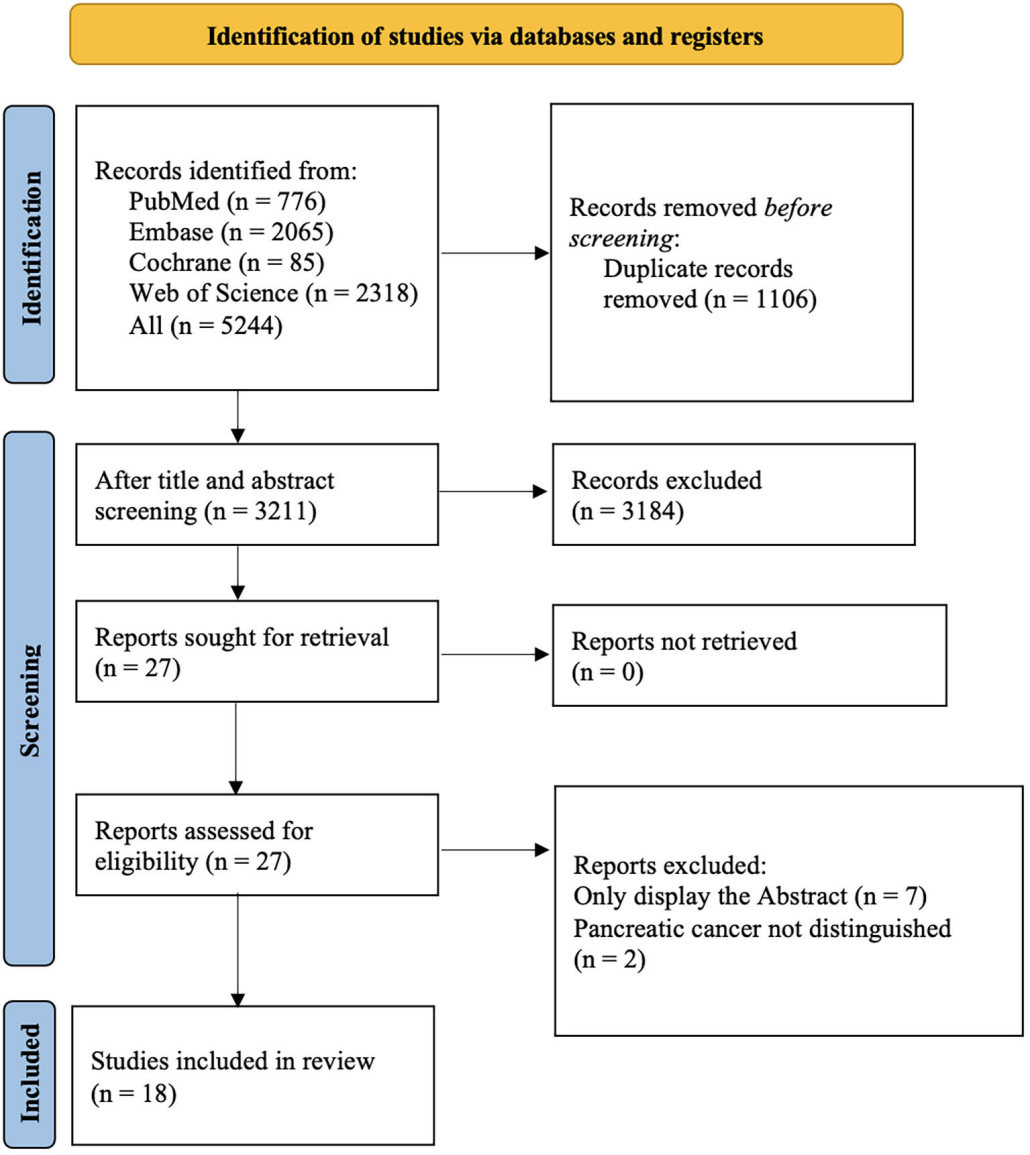
The literature screening process.

Among 5,160 retrieved records, 1,106 were duplicate studies. After screening based on the titles and abstracts, 3211 studies were selected. Abstracts with incomplete information were excluded, and ultimately, 18 studies were selected (**Figure 1**).

### 3.2 Characteristics of the studies

Among the 18 studies selected, 1 was a prospective cohort study, 2 adopted a case-control design, 2 were nested case-control population-based studies, 1 was a retrospective observational study, and the remaining 12 were all retrospective cohort studies. A total of 18 studies investigated the risk factors for PC in patients with DM, and 13 of these studies established prediction models for PC among DM patients employing ML algorithms. The data used in the studies mainly came from various databases, including the UK Biobank database in 2 studies, TriNetX database in 3 studies, and regional single-center/multi-center data from research sites in 13 studies. The average follow-up period of those studies was around 3 years. The ML algorithms used in the studies included LR, SVM, MLP, XG Boost, RF, PRS, Boursi model, iterative linearization method, ANN, neural network, LDA, Light GBM, GBM, SVC, Voting, END-PAC scoring model, and Cox model. The selected modeling variables all had clinical characteristics. In a study, variables related to genetic features were also included. To verify the performance of the models, these studies adopted internal validation (random sampling, K-fold cross-validation, Bootstrap), and some also employed external validation (**Table 1**).

**Table 1:** Basic information of the selected literature.

No.	The first author	The publication period	Author country	Study type	Patient source	Follow-up duration	Number of pancreatic cancer	Total number of cases	Prediction model	Verification set generation method	Type of model
1	Yongji Sun	2024	China	Prospective cohort study	UK Biobank	2 years	100	12,635	YES	5-fold cross-validation	LR/SVM/MLP /XGBoost/RF
2	Shreya Sharma	2022	UK	Nested case-control population-based study	UK Biobank	24 months	1,042	104,306	YES		PRS
3	Ayush Sharma	2018	USA	Retrospective cohort study	The rochester epidemiology project	3 years	64	1,561	YES	External verification	LR
4	Salman Khan	2021	USA	Retrospective case-control study	TriNetX	4 years	48	6,302	YES		END-PAC model
5	Salman Khan	2024	USA	Retrospective cohort study	TriNetX	1 year	380	81,213	YES	10-fold cross-validation	XGBoost
6	Salman Khan	2021	USA	Retrospective cohort study	TriNetX	3 years	52	27,945	YES		Boursi model
7	Christie Y. Jeon	2022	USA	Retrospective cohort study	Veterans affairs health system	12, 36, and 60 months	6,300	799,529	YES	Bootstrap	Cox
8	Meng hsuen hsieh	2018	Taiwan, China	Retrospective cohort study	The National Health Insurance Research Database (NHIRD) of Taiwan	The average follow-up period for the pancreatic cancer group was 3.84 years, while that for the non-pancreatic cancer group was 6.87 years.	3,092	1,358,634	YES	10-fold cross-validation	LR/ANN
9	Ash Kieran Clift	2024	UK	Retrospective cohort study	QResearch database	2 years	767	253,766	YES	Internal and external cross-validation	Cox/XGBoost/ANN
10	Simon Lebech Cichosz	2023	Denmark	Retrospective cohort study	The Danish National Patient Registry, Danish National Prescription Registry, and Civil Registration System	3 years	716	353,970	YES	Random sampling	RF
11	Shih-MinChen	2023	Taiwan, China	Retrospective observational study	Taipei Medical University Clinical Research Database	4 years	89	66,384	YES	5-fold cross-validation method	LR/LDA/RF/LightGBM/GBM/XGB/SVM
12	Ben Boursi	2022	Israel	Retrospective cohort study	Maccabi Health care Services	3 years	59	5,408	YES		END-PAC model
13	Sitwat Ali	2024	Australia	Retrospective cohort study	Australian administrative health databases	3 years	602	99,687	YES	Random sampling	LR
14	Omid Sadr-Azodi	2015	Sweden	Nested case-control population-based study	The Swedish National Diabetes Register	5 years	391	4,301	NO		
15	Satish Munigala	2015	USA	Retrospective cohort study	The Veterans Health Administration national medical care data	3 years	183	73,811	NO		
16	Suguru Mizuno	2012	Japan	Case–control study	single-center		40	160	NO		
17	Raymond Ngai Chiu Chan	2022	China	Retrospective cohort study	Clinical Data Analysis and Reporting System	3547 ± 1207(d)	1,148	273,738	NO		
18	Tatsunori Satoh	2024	Japan	Retrospective cohort study	Shizuoka Kokuho database	9.5 years	1,755	212,775	NO		

### 3.3 Risk of bias in studies

The NOS scale was employed to assess the quality of the studies selected. Among the 18 studies selected, 1 was a prospective cohort study, 2 were case-control studies, 2 were nested case-control population-based studies, 1 was a retrospective observational study, and the remaining 12 were all retrospective cohort studies. Among them, only one study was conducted based on a specific group, such as veterans. Therefore, it did not receive any points for the representativeness of the exposure cohort. There were ten studies with a follow-up period of no more than three years. We believed that such a duration might lead to insufficient follow-up, and therefore, it did not receive any points either. In addition, all the 18 studies received 2 points each in the comparability analysis item, and 1 point each in other items. The final scores of the 18 studies selected were all between 8 and 9, featuring all high-quality studies.

### 3.4 Risk factors

In the studies selected, 6 studies used the Cox regression method, 7 employed the logistic regression approach to investigate the risk factors for PC among patients with DM, and the remaining 5 discussed the modeling variables providing a higher predictive effect in the constructed prediction models. Due to the diversity of risk factors and potential differences in the assessment methods among various factors, an overview of them was only provided (**Table 2**).

**Table 2:** Risk factors for pancreatic cancer (PC) in patients with diabetes mellitus (DM) in various studies.

No.	The first author	Model	Risk factors
1	Yongji Sun	Logistic regression analysis	Incorporating age at recruitment, platelet count, systolic blood pressure, immature reticulocyte fraction, platelet crit, 24 single nucleotide polymorphisms
2	Shreya Sharma	Cox regression	Ancestry, smoking, DM, waist circumference, and family history of digestive cancer
3	Ayush Sharma	Logistic regression analysis	Change in weight, change in blood glucose, age at onset of DM
4	Salman Khan		Age at diabetes diagnosis, BMI, HbAlc, History of pancreatitis, smoking, and obesity
5	Salman Khan	Shapley Additive explanation	Age at diabetes diagnosis, weight loss, ΔGlucose (the change in glucose over the year preceding diabetes onset);laboratory values: HbA1C; alkaline phosphatase; hemoglobin; anemic prescribed anti-diabetic medicines (insulin, metformin, or oral anti-glycemic agents) and proton pump inhibitors.
6	Salman Khan	Regression coefficients	HbA1c, alkaline phosphatase, hemoglobin, creatinine, total cholesterol, insulin, metformin, oral antidiabetic drugs, and PPIs
7	Christie Y. Jeon	Cox proportional hazards regression	Age, current smoking, weight increase, acute pancreatitis, chronic pancreatitis, abdominal pain, jaundice, bilirubin
8	Meng hsuen hsieh		Age, gender, acute pancreatitis, chronic pancreatitis, gallstone, cholecystectomy, cirrhosis, hyperlipidemia, obesity, CCi score, retinopathy, nephropathy, neuropathy, cerebrovascular, peripheral vascular disease, statin, sulfonylureas, TZD, other antidiabetic drugs, adapted Diabetes Complication Severity Index (aDCSI)
9	Ash Kieran Clift	Cox proportional hazards modelling	Age, gender, BMI, previous venous thromboembolism, HbA1c, ALT, creatinine, hemoglobin, platelet count; recent digoxin use, recent Abdominal pain, recent weight loss, recent heartburn, recent indigestion, recent nausea, jaundice
10	Simon Lebech Cichosz	RR value	Age, gender, HbAlc, Triglycerides, HDL, LDL, Bilirubin, Alkaline Phosphatase, ALAT, GGT, INR, Haemoglobin, Albumin, CRP, Leukocytes, Platelets
11	Shih-MinChen	Logistic regression analysis	Glucose, glycated hemoglobin, hyperlipidemia comorbidity, antidiabetic drug use, and lipid modifying drug use
12	Ben Boursi	Cox proportion hazard model	Age at index date, change in body weight, and change in FPG
13	Sitwat Ali	Logistic regression analysis	Age at diabetes diagnosis, severity of diabetes, and use of insulin, beta-blockers, acid-disrupting drugs, and lipid-modulating agents
14	Omid Sadr-Azodi	Logistic regression analysis	Glycated hemoglobin (HbA1c) change
15	Satish Munigala	Logistic regression analysis	Non-obese (OR 1.51; 95% CI 1.14-1.99; P = 0.0035), Age>65 years (OR 2.01; 95% CI 1.51-2.68, P< 0.0001), heavy smokers (OR 1.55; 95% CI 1.12-2.14, P = 0.009), history of CP (OR 4.72; 95% CI 2.71-8.24, P< 0.0001), Gallstones (OR 2.02; 95%CI 1.32-3.11, P = 0.0013).
16	Suguru Mizuno	Logistic regression analysis	In patients with early-onset DM, family history of DM (OR 3.60; 95% CI 1.03–15.09; P = 0.04);use of insulin (OR 3.52; 95% CI 1.00–14.87; P = 0.05); duration and dose of insulin (ORs 4.77; 95% CI 1.09–22.34; P = 0.04); In patients with late-onset DM, DM-onset age (OR 1.12; 95% CI 1.03–1.24; P<0.01);multiple diabetic patients in the family (OR 6.13; 95% CI 1.20–37.91;P = 0.03).
17	Raymond Ngai Chiu Chan	Cox regression analysis	Pancreatic diseases (HR: 32.68;95% CI, 18.05-59.18; P< 0.001); CKD (HR: 1.70;95% CI, 1.36-2.12; P< 0.001); Patients withless than stage 2 CKD (HR: 1.91; 95% CI, 1.28-2.83; P = 0.001); ALP(HR: 1.00;95% CI, 1.00-1.01;P = 0.040); GLPA (HR: 2.67; 95% CI, 1.00-7.12; P = 0.050); alpha-glucosidase inhibitor (HR: 1.76; 95% CI, 1.16-2.65; P = 0.008)
18	Tatsunori Satoh	Cox regression analysis	older age and male sex (HR: 1.13, 95% CI 1.01–1.26), coexisting liver disease (HR:1.13, 95% CI 1.01-1.26), chronic pancreatitis (HR:1.98, 95% CI 1.48-2.64; P<0.001), and pancreatic cystic lesions (HR:4.79, 95% CI 3.43-6.67; P<0.001)

#### 3.4.1 Clinical characteristics

The increased risk of PC among patients with DM may be associated to older age, pancreatic-related diseases (such as chronic pancreatitis and pancreatic cystic lesions), history of liver diseases (such as non-alcoholic fatty liver disease (NAFLD)), jaundice, gallstones, and chronic kidney disease (CKD) at a stage less than 2 (13–17). It is worth noting that the risk of developing new-onset DM is greater than that of pre-existing DM. Patients with this condition present with weight loss and rapid deterioration of glycemic control within 1–2 years before the diagnosis of DM (13, 18). When patients with new-onset DM experience symptoms such as abdominal pain, nausea, and vomiting, this may indicate the possibility of PC (14).

#### 3.4.2 Biochemical indicators

In terms of biochemical indicators, certain differences in lipid metabolism and liver function are also observed between patients with new-onset DM and those with pre-existing DM. These include variations in triglyceride (TG), alkaline phosphatase (ALP), and alanine aminotransferase (ALT) (14). A higher rate of HbA1c alteration and elevated ALP are correlated with the occurrence of PC (19). Notably, alterations in these biochemical indicators may be the consequence of early-stage PC. Therefore, they could potentially be used in clinical practice to identify high-risk populations for early-stage PC.

#### 3.4.3 Medication history

During the DM treatment process, changes in the timing of insulin administration, alterations in the medication regimen, and the application of new medications may be associated with the onset of PC (20). The initiation of insulin use, metformin, and other oral hypoglycemic drugs is correlated with a heightened risk of PC (19, 20). The use of GLP-1RAs, a novel class of antidiabetic drugs in clinical practice in recent years, has been observed to be associated with a change in the incidence of PC among treated patients (16). Additionally, some drugs used to treat complications of DM (such as β-blockers, acid-suppressing drugs, and lipid-regulating medications) are also associated with the risk of PC (19, 21). Nevertheless, as the discussion on the role of medication history in the onset of PC in the original studies included remains limited, the observed association between drug exposure and PC should be interpreted with caution.

During the course of these studies, due to ethnicity, region, scale, educational level, and information deficiency, certain deviations may exist in the prediction results. However, the early identification of the risk factors above is still of great significance for predicting the occurrence of PC, assisting in the early treatment of PC, and improving the survival of patients with PC. Early surgical treatment is currently the most effective means to strive for the survival of PC patients. Efforts should be made to develop powerful tools to detect high-risk populations for PC, in order to provide accurate and reliable clinical prediction tools for the prevention and treatment of PC.

### 3.5 Meta-analysis

#### 3.5.1 Incidence rate

**Figure 2:**
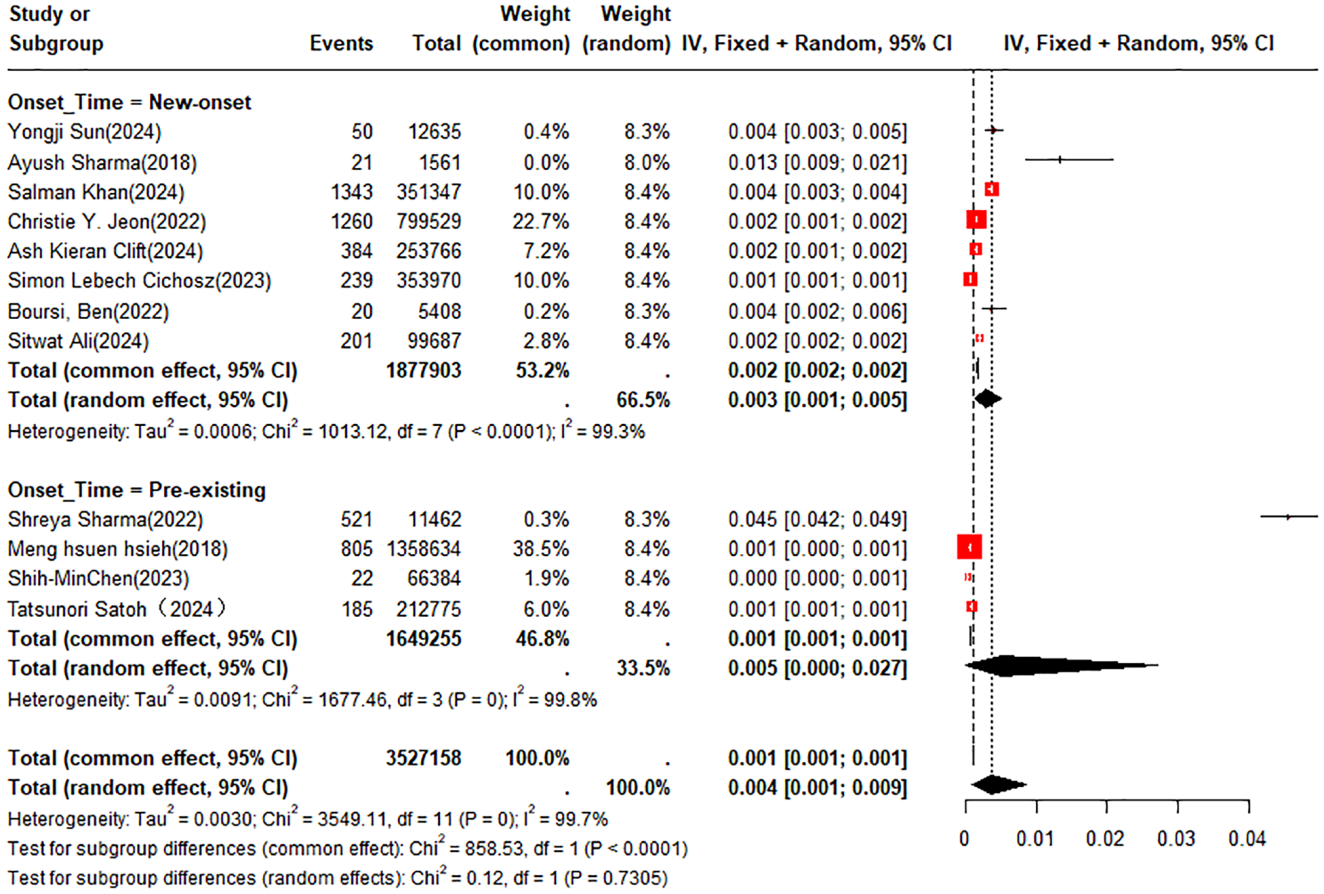
Meta-analysis forest plot of annual incidence rate in new-onset DM vs. pre-existing DM.

**Figure 3:**
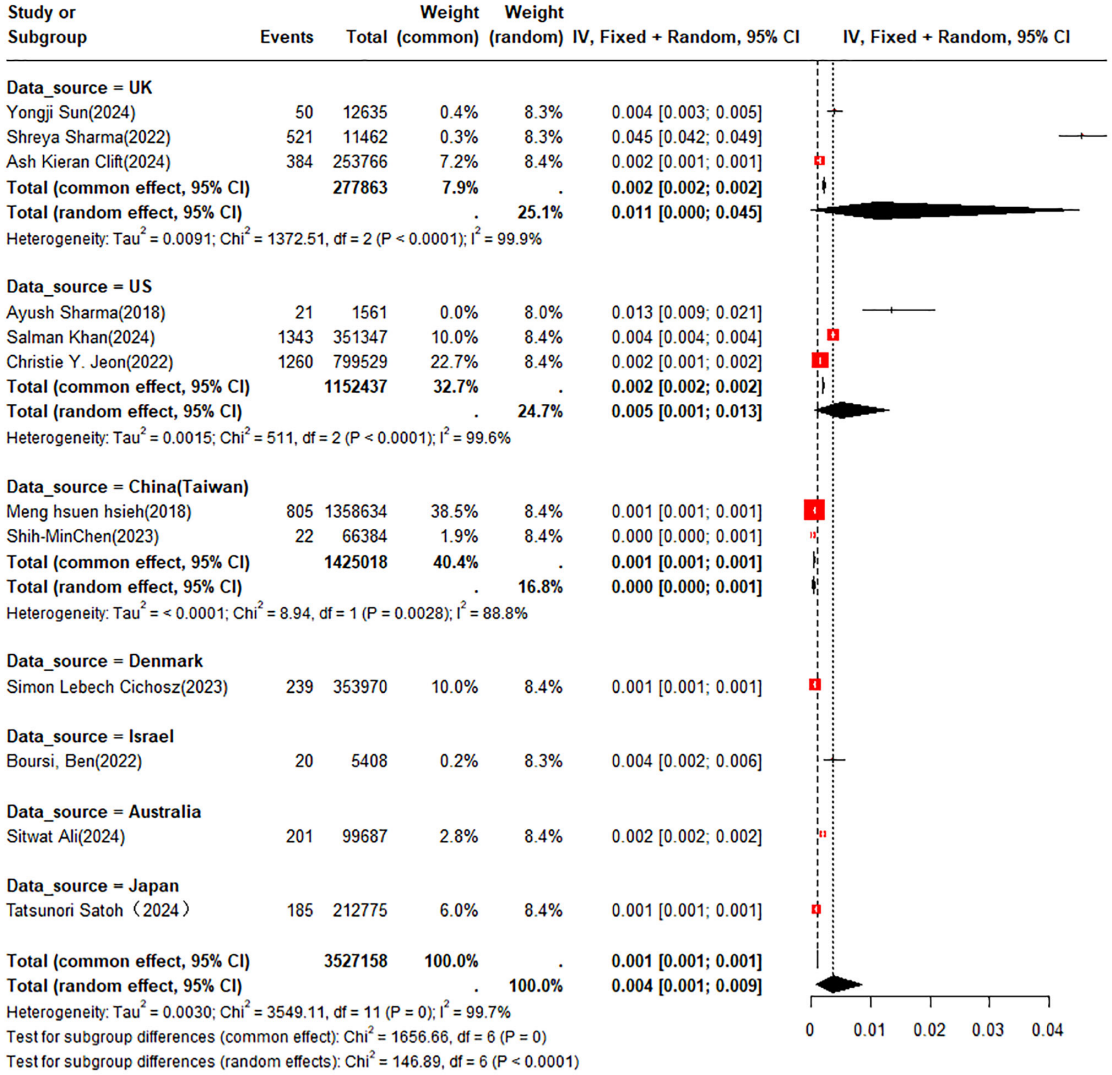
Meta-analysis forest plot for annual incidence rate by country/region.

A double arcsine transformation was applied prior to meta-analysis of the 18 included studies. A random-effects model was utilized to summarize the average annual incidence rate, which was found to be approximately 0.4% (95% CI: 0.1% - 0.9%). Subgroup analyses of incidence rates were performed according to new-onset DM or pre-existing DM and country or region. The incidence rates of PC were 0.3% (95% CI: 0.1% - 0.5%) in patients with new-onset DM and 0.5% (95% CI: 0% - 2.7%) in those with pre-existing DM, respectively (**Figure 2**). In the subgroup analysis by country or region, the incidence rate of PC was 1.1% (95% CI: 0% - 4.5%) in patients from the UK compared to 0.5% (95% CI: 0.1% - 1.3%) in those from the U.S. (**Figure 3**).

#### 3.5.2 Accuracy of the prediction model

**Figure 4:**
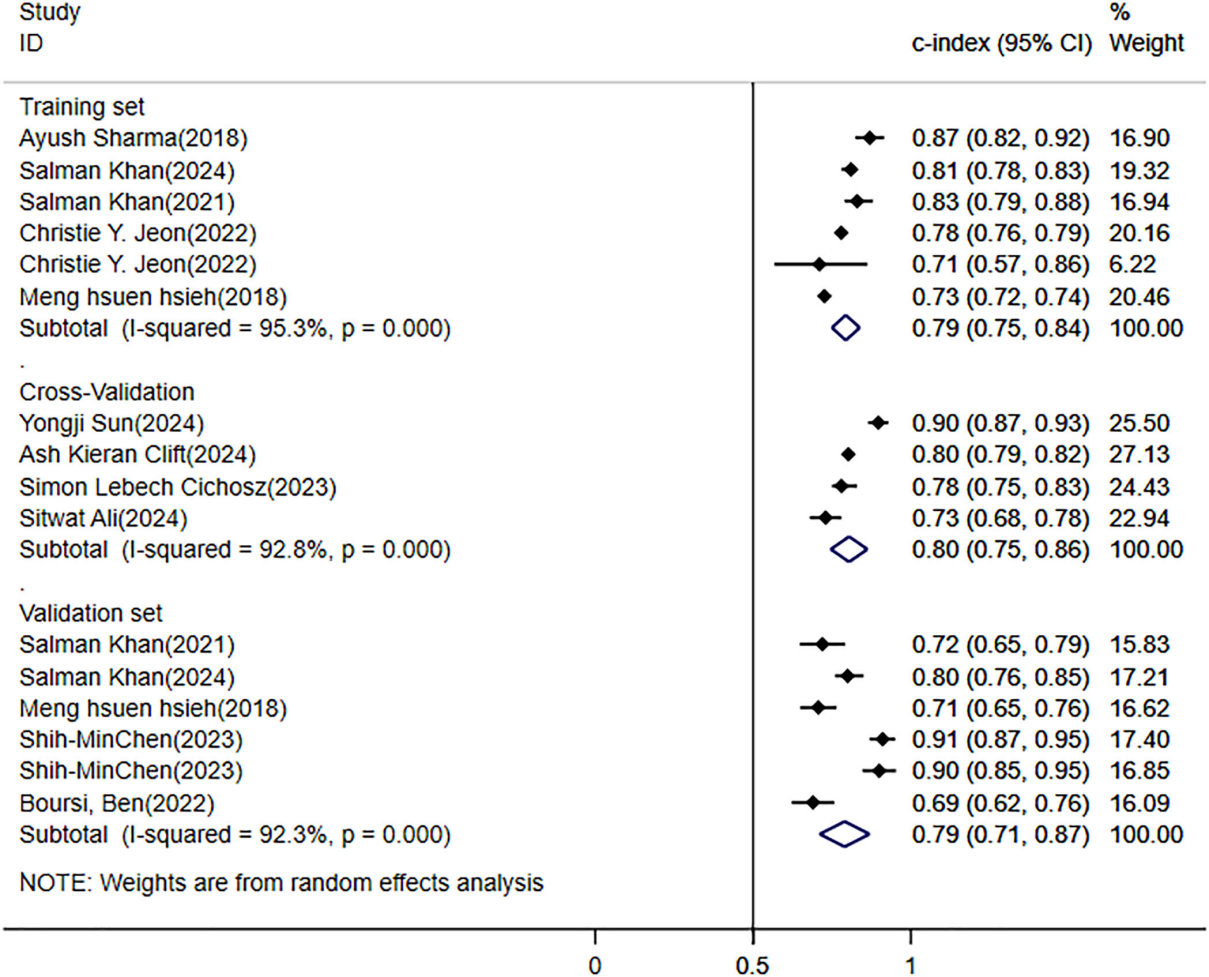
Meta-analysis forest plot of the C-Index for pancreatic cancer (PC) risk prediction model in patients with diabetes mellitus (DM).

Among the literature included in this study, 5 provided the c-index of the training set, 4 conducted cross-validation, and 5 verified the models built on the training set. The results based on the random effects model indicated that the c-index of the training set was 0.79 (95% CI: 0.75 - 0.84, I^2^ = 95.3%); the c-index of the validation set was 0.79 (95% CI: 0.71 - 0.87, I^2^ = 92.3%); and the c-index of the cross-validation was 0.80 (95% CI: 0.75 - 0.86, I^2^ = 92.8%) (**Figure 4**).

## 4 Discussion

### 4.1 Summary of the main findings

In the systematic review, the annual incidence rate of PC among diabetic patients was 0.4% (95% CI: 0.1% - 0.9%). Through meta-analysis, in the current situation where there are no efficient prediction tools, constructing a prediction model for the risk of PC using ML methods seems to be a feasible clinical prediction approach. The aggregated c-index of the training set and the validation set was 0.79 (95% CI: 0.75 - 0.84, I^2^ = 95.3%) and 0.79 (95% CI: 0.71 - 0.87, I^2^ = 92.3%), respectively. Among the 18 studies selected, there existed diverse risk factors.

### 4.2 Comparison with previous reviews

Patients with DM tend to have an elevated risk of PC. A meta-analysis by Qiwen Ben et al. has confirmed that DM is an independent risk factor for PC (the summary RRs = 1.94; 95% CI: 1.66 - 2.27) (7). This study also confirmed this view. In this study, the annual average incidence rate of PC among patients with DM was 0.4% (95% CI: 0.1% - 0.9%). The incidence rates of PC among patients with new-onset DM and those with pre-existing DM were 0.3% (95% CI: 0.1% - 0.5%) and 0.5% (95% CI: 0% - 2.7%), respectively. The incidence of PC was higher in patients suffering from pre-existing DM compared with those experiencing new-onset DM. Nonetheless, the wide confidence interval (0% - 2.7%) suggested that this might be related to the heterogeneity of the study population. The results for the group of patients with new-onset DM were more concentrated and stable (0.1% - 0.5%), signifying that new-onset DM was a warning sign for PC. These findings emphasize the vital significance of integrating PC screening and prevention into the clinical management of DM. However, current strategies are hindered by the lack of reliable risk prediction tools. Existing literature in this domain has chiefly focused on identifying risk factors relative to PC to guide targeted prevention strategies. While the intended purpose is to accurately identify target patients, challenges remain in achieving effective early diagnosis of PC, and the accuracy of existing prediction models remains suboptimal. Moreover, early diagnosis of PC is critical for therapeutic outcomes, yet during the process of model establishment, the lack of stage-specific diagnostic data limits their applicability for predicting early-stage PC (21).

The progression of DM to complications is influenced by multiple factors. In addition to evaluating the performance of predictive models in included studies, our review synthesizes evidence on risk factors for PC development in patients with DM. Previous studies have established DM as an independent risk factor for PC (7), while others have identified additional contributors, including advanced age, smoking, alcohol consumption, family history, and pancreatitis (8, 22). However, these studies offer only a limited exploration of DM-related risk factors that may induce PC, which may compromise the establishment and interpretation of clinical prediction models. The primary objective of current research in this field is to identify robust risk factors to facilitate early intervention in high-risk individuals. Our study systematically reviews risk factors reported in the included literature, providing evidence-based recommendations for the future development of intelligent prediction tools.

### 4.3 Challenges faced by prediction model

Based on our synthesized findings, ML-based models have demonstrated favorable accuracy in predicting PC risk among DM patients. However, several challenges persist in their development and application.

Firstly, the low annual incidence of PC in modeling datasets results in severe imbalance. None of the included studies addressed data imbalance during model construction, and only a few reported sensitivity and specificity. That forces us to question the accuracy of the prediction results, as they may be influenced by a higher number of negative events. Therefore, there are still challenges in screening for positive events, such as the early diagnosis of PC. Therefore, future studies should account for the impact of imbalanced data on the construction of ML models. During the model construction process, it is advisable to attempt to use balanced data to build models, and then validate them using imbalanced data. Additionally, comprehensive evaluation metrics should be provided to assist in improving the model performance as much as possible, thereby reducing the impact of imbalanced data.

Secondly, variations in follow-up duration may influence model accuracy. Shorter follow-up periods could lead to underrepresentation of PC cases. That results in a lack of comprehensive evidence for PC, thereby limiting the accuracy of prediction models. Among the included studies, follow-up durations varied, making it difficult for us to discuss the predictive accuracy of the models. Future research should rigorously consider the accuracy of the models during different follow-up periods, and promptly update the models for each follow-up period.

Thirdly, the choice of ML algorithms affects both predictive performance and accuracy. When selecting models, we need to balance interpretability with predictive accuracy. Interpretability is one of the attributes of ML. During the modeling stage, the higher the interpretability, the more it helps people understand why such predictions are made. In clinical practice, when physicians utilize prediction models, they can employ medical terminology to clearly explain the model’s underlying mechanisms. This enhances both model transparency and patient comprehension, thereby strengthening trust in both the medical professionals and the prediction models. Ultimately, this facilitates the orderly implementation of evidence-based clinical work. Models with better interpretability, such as logistic regression, Cox regression, decision trees, and linear discriminant functions, often demonstrate poorer accuracy due to their inability to handle more complex relationships. In contrast, models capable of high-precision processing, such as random forests and XGBoost, achieve higher accuracy, but their complex internal mechanisms may lead to a “black box” phenomenon (23). Regarding the processing of imbalanced data, the interpretability and the accuracy are primarily attributed to the negative events.

### 4.4 Deviation from protocol

We ultimately employed the NOS scale to evaluate the bias risk in the studies selected, rather than the PROBAST tool specified in the study protocol. This decision was made because the included literature comprised not only studies developing or validating prediction models but also a subset of articles solely examining risk factors without model construction. Given this heterogeneity in study design, the NOS was deemed more suitable for a unified quality assessment across all eligible literature.

## 5 Strengths and limitations

### 5.1 Strengths

This study pioneers the comprehensive evaluation of the predictive value of ML models for identifying PC risk in patients with DM. We further discuss the current applications and limitations of such models, providing evidence-based insights to guide the development and refinement of future predictive tools.

### 5.2 Limitations

Firstly, the number of eligible studies after systematic retrieval is limited, and the investigated prediction models exhibited relative homogeneity in methodology. Secondly, the models were primarily trained on imbalanced datasets, yet none of the included studies addressed potential bias arising from severe class imbalance, which may compromise the reported accuracy in predicting PC among diabetic patients. Thirdly, most studies derived their data from overlapping databases, resulting in restricted geographic and ethnic representation, thereby limiting the generalizability of our findings. Fourthly, there are still certain limitations in the analysis of incidence rates. Since the original studies included only provided follow-up time and failed to discuss the incidence rate of PC in terms of the course of DM, stratified analyses of the incidence rate of PC among patients with DM at different disease courses were not carried out. Lastly, the discussion on risk factors such as drug exposure remained restricted.

## 6 Conclusion

ML-based prediction models demonstrate favorable value in predicting PC risk among patients with DM. Future research could leverage these approaches to dynamically update prediction algorithms. However, the studies included in our analysis predominantly relied on severely imbalanced datasets for model development, with limited discussion on the impact of follow-up duration on predictive performance. Therefore, subsequent studies should lay emphasis on larger and more representative patient cohorts, construct more widely applicable models, and dynamically update ML models.

## Data Availability

The original contributions presented in the study are included in the article/**Supplementary Material**. Further inquiries can be directed to the corresponding author.
