# Supplementary material for: Risk factors and early prediction of pancreatic cancer among patients with diabetes mellitus: a systematic review and meta-analysis
Source: Front Endocrinol (Lausanne). 2025 Nov 19;16:1698850. doi: 10.3389/fendo.2025.1698850 (PMC12673663; doi:10.3389/fendo.2025.1698850)
Supplement: Supplementary file 1 [file DataSheet1.docx]

Additional Material 2 Literature search strategy

**1.Pubmed**

| Search number | Query | Results |
| --- | --- | --- |
| 1 | "Diabetes Mellitus"[Mesh] | 538,225 |
| 2 | (((((((Diabetes Mellitus[Title/Abstract]) OR (Type 2 Diabetes[Title/Abstract])) OR (T2DM[Title/Abstract])) OR (T1DM[Title/Abstract])) OR (Type 1 Diabetes[Title/Abstract])) OR (Diabetes[Title/Abstract])) OR (diabetic[Title/Abstract])) OR (diabets[Title/Abstract]) | 838,884 |
| 3 | ("Diabetes Mellitus"[Mesh]) OR ((((((((Diabetes Mellitus[Title/Abstract]) OR (Type 2 Diabetes[Title/Abstract])) OR (T2DM[Title/Abstract])) OR (T1DM[Title/Abstract])) OR (Type 1 Diabetes[Title/Abstract])) OR (Diabetes[Title/Abstract])) OR (diabetic[Title/Abstract])) OR (diabets[Title/Abstract])) | 905,899 |
| 4 | machine learning[MeSH Terms] | 80,357 |
| 5 | (((((((((((((((((((((((((((((((((((((machine learning[Title/Abstract]) OR (risk factors[Title/Abstract])) OR (predictors[Title/Abstract])) OR (biomarkers[Title/Abstract])) OR (prediction model[Title/Abstract])) OR (risk model[Title/Abstract])) OR (risk score[Title/Abstract])) OR (artificial intelligence[Title/Abstract])) OR (Transfer Learning[Title/Abstract])) OR (Deep learning[Title/Abstract])) OR (Ensemble Learning[Title/Abstract])) OR (random forest[Title/Abstract])) OR (neural network[Title/Abstract])) OR (neural networks[Title/Abstract])) OR (CNN[Title/Abstract])) OR (K-Nearest Neighbor[Title/Abstract])) OR (Support vector machine[Title/Abstract])) OR (SVM[Title/Abstract])) OR (Gradient Boosting Machine[Title/Abstract])) OR (Nomogram[Title/Abstract])) OR (XGBoost[Title/Abstract])) OR (Adaboost[Title/Abstract])) OR (LightGBM[Title/Abstract])) OR (CatBoost[Title/Abstract])) OR (Gradient Boosting[Title/Abstract])) OR (Decision tree[Title/Abstract])) OR (Regression Trees[Title/Abstract])) OR (ResNet[Title/Abstract])) OR (AlexNet[Title/Abstract])) OR (VGGNet[Title/Abstract])) OR (GoogLeNet[Title/Abstract])) OR (Naive Bayesian[Title/Abstract])) OR (Multilayer perceptron[Title/Abstract])) OR (Bayesian network[Title/Abstract])) OR (Radiomics[Title/Abstract])) OR (Radiomic[Title/Abstract])) OR (radiomics-based[Title/Abstract])) OR (Texture[Title/Abstract]) | 1,653,887 |
| 6 | (machine learning[MeSH Terms]) OR ((((((((((((((((((((((((((((((((((((((machine learning[Title/Abstract]) OR (risk factors[Title/Abstract])) OR (predictors[Title/Abstract])) OR (biomarkers[Title/Abstract])) OR (prediction model[Title/Abstract])) OR (risk model[Title/Abstract])) OR (risk score[Title/Abstract])) OR (artificial intelligence[Title/Abstract])) OR (Transfer Learning[Title/Abstract])) OR (Deep learning[Title/Abstract])) OR (Ensemble Learning[Title/Abstract])) OR (random forest[Title/Abstract])) OR (neural network[Title/Abstract])) OR (neural networks[Title/Abstract])) OR (CNN[Title/Abstract])) OR (K-Nearest Neighbor[Title/Abstract])) OR (Support vector machine[Title/Abstract])) OR (SVM[Title/Abstract])) OR (Gradient Boosting Machine[Title/Abstract])) OR (Nomogram[Title/Abstract])) OR (XGBoost[Title/Abstract])) OR (Adaboost[Title/Abstract])) OR (LightGBM[Title/Abstract])) OR (CatBoost[Title/Abstract])) OR (Gradient Boosting[Title/Abstract])) OR (Decision tree[Title/Abstract])) OR (Regression Trees[Title/Abstract])) OR (ResNet[Title/Abstract])) OR (AlexNet[Title/Abstract])) OR (VGGNet[Title/Abstract])) OR (GoogLeNet[Title/Abstract])) OR (Naive Bayesian[Title/Abstract])) OR (Multilayer perceptron[Title/Abstract])) OR (Bayesian network[Title/Abstract])) OR (Radiomics[Title/Abstract])) OR (Radiomic[Title/Abstract])) OR (radiomics-based[Title/Abstract])) OR (Texture[Title/Abstract])) | 1,659,209 |
| 7 | Pancreatic Neoplasms[MeSH Terms] | 96,849 |
| 8 | (((((((((((((((((((((((((Pancreatic Neoplasms[Title/Abstract]) OR (Pancreatic Neoplasm[Title/Abstract])) OR (Pancreas Neoplasms[Title/Abstract])) OR (Pancreas Neoplasm[Title/Abstract])) OR (Cancer of Pancreas[Title/Abstract])) OR (Pancreas Cancers[Title/Abstract])) OR (Pancreas Cancer[Title/Abstract])) OR (Pancreatic Cancer[Title/Abstract])) OR (Pancreatic Cancers[Title/Abstract])) OR (Cancer of the Pancreas[Title/Abstract])) OR (Islet Cell Carcinoma[Title/Abstract])) OR (Islet Cell Carcinomas[Title/Abstract])) OR (Pancreatic Intraductal Neoplasms[Title/Abstract])) OR (Pancreatic Intraductal Neoplasm[Title/Abstract])) OR (Pancreatic IPMN[Title/Abstract])) OR (Pancreatic IPMNs[Title/Abstract])) OR (Pancreatic Ductal Carcinomas[Title/Abstract])) OR (Duct-Cell Carcinoma of the Pancreas[Title/Abstract])) OR (Duct Cell Carcinoma of the Pancreas[Title/Abstract])) OR (Pancreatic Ductal Carcinoma[Title/Abstract])) OR (Ductal Carcinoma of the Pancreas[Title/Abstract])) OR (Pancreatic Duct Cell Carcinoma[Title/Abstract])) OR (Pancreas Duct-Cell Carcinoma[Title/Abstract])) OR (Pancreas Duct-Cell Carcinomas[Title/Abstract])) OR (pancreas tumor[Title/Abstract])) OR (pancreas tumour[Title/Abstract]) | 65,179 |
| 9 | (Pancreatic Neoplasms[MeSH Terms]) OR ((((((((((((((((((((((((((Pancreatic Neoplasms[Title/Abstract]) OR (Pancreatic Neoplasm[Title/Abstract])) OR (Pancreas Neoplasms[Title/Abstract])) OR (Pancreas Neoplasm[Title/Abstract])) OR (Cancer of Pancreas[Title/Abstract])) OR (Pancreas Cancers[Title/Abstract])) OR (Pancreas Cancer[Title/Abstract])) OR (Pancreatic Cancer[Title/Abstract])) OR (Pancreatic Cancers[Title/Abstract])) OR (Cancer of the Pancreas[Title/Abstract])) OR (Islet Cell Carcinoma[Title/Abstract])) OR (Islet Cell Carcinomas[Title/Abstract])) OR (Pancreatic Intraductal Neoplasms[Title/Abstract])) OR (Pancreatic Intraductal Neoplasm[Title/Abstract])) OR (Pancreatic IPMN[Title/Abstract])) OR (Pancreatic IPMNs[Title/Abstract])) OR (Pancreatic Ductal Carcinomas[Title/Abstract])) OR (Duct-Cell Carcinoma of the Pancreas[Title/Abstract])) OR (Duct Cell Carcinoma of the Pancreas[Title/Abstract])) OR (Pancreatic Ductal Carcinoma[Title/Abstract])) OR (Ductal Carcinoma of the Pancreas[Title/Abstract])) OR (Pancreatic Duct Cell Carcinoma[Title/Abstract])) OR (Pancreas Duct-Cell Carcinoma[Title/Abstract])) OR (Pancreas Duct-Cell Carcinomas[Title/Abstract])) OR (pancreas tumor[Title/Abstract])) OR (pancreas tumour[Title/Abstract])) | 116,932 |
| 10 | ((("Diabetes Mellitus"[Mesh]) OR ((((((((Diabetes Mellitus[Title/Abstract]) OR (Type 2 Diabetes[Title/Abstract])) OR (T2DM[Title/Abstract])) OR (T1DM[Title/Abstract])) OR (Type 1 Diabetes[Title/Abstract])) OR (Diabetes[Title/Abstract])) OR (diabetic[Title/Abstract])) OR (diabets[Title/Abstract]))) AND ((machine learning[MeSH Terms]) OR ((((((((((((((((((((((((((((((((((((((machine learning[Title/Abstract]) OR (risk factors[Title/Abstract])) OR (predictors[Title/Abstract])) OR (biomarkers[Title/Abstract])) OR (prediction model[Title/Abstract])) OR (risk model[Title/Abstract])) OR (risk score[Title/Abstract])) OR (artificial intelligence[Title/Abstract])) OR (Transfer Learning[Title/Abstract])) OR (Deep learning[Title/Abstract])) OR (Ensemble Learning[Title/Abstract])) OR (random forest[Title/Abstract])) OR (neural network[Title/Abstract])) OR (neural networks[Title/Abstract])) OR (CNN[Title/Abstract])) OR (K-Nearest Neighbor[Title/Abstract])) OR (Support vector machine[Title/Abstract])) OR (SVM[Title/Abstract])) OR (Gradient Boosting Machine[Title/Abstract])) OR (Nomogram[Title/Abstract])) OR (XGBoost[Title/Abstract])) OR (Adaboost[Title/Abstract])) OR (LightGBM[Title/Abstract])) OR (CatBoost[Title/Abstract])) OR (Gradient Boosting[Title/Abstract])) OR (Decision tree[Title/Abstract])) OR (Regression Trees[Title/Abstract])) OR (ResNet[Title/Abstract])) OR (AlexNet[Title/Abstract])) OR (VGGNet[Title/Abstract])) OR (GoogLeNet[Title/Abstract])) OR (Naive Bayesian[Title/Abstract])) OR (Multilayer perceptron[Title/Abstract])) OR (Bayesian network[Title/Abstract])) OR (Radiomics[Title/Abstract])) OR (Radiomic[Title/Abstract])) OR (radiomics-based[Title/Abstract])) OR (Texture[Title/Abstract])))) AND ((Pancreatic Neoplasms[MeSH Terms]) OR ((((((((((((((((((((((((((Pancreatic Neoplasms[Title/Abstract]) OR (Pancreatic Neoplasm[Title/Abstract])) OR (Pancreas Neoplasms[Title/Abstract])) OR (Pancreas Neoplasm[Title/Abstract])) OR (Cancer of Pancreas[Title/Abstract])) OR (Pancreas Cancers[Title/Abstract])) OR (Pancreas Cancer[Title/Abstract])) OR (Pancreatic Cancer[Title/Abstract])) OR (Pancreatic Cancers[Title/Abstract])) OR (Cancer of the Pancreas[Title/Abstract])) OR (Islet Cell Carcinoma[Title/Abstract])) OR (Islet Cell Carcinomas[Title/Abstract])) OR (Pancreatic Intraductal Neoplasms[Title/Abstract])) OR (Pancreatic Intraductal Neoplasm[Title/Abstract])) OR (Pancreatic IPMN[Title/Abstract])) OR (Pancreatic IPMNs[Title/Abstract])) OR (Pancreatic Ductal Carcinomas[Title/Abstract])) OR (Duct-Cell Carcinoma of the Pancreas[Title/Abstract])) OR (Duct Cell Carcinoma of the Pancreas[Title/Abstract])) OR (Pancreatic Ductal Carcinoma[Title/Abstract])) OR (Ductal Carcinoma of the Pancreas[Title/Abstract])) OR (Pancreatic Duct Cell Carcinoma[Title/Abstract])) OR (Pancreas Duct-Cell Carcinoma[Title/Abstract])) OR (Pancreas Duct-Cell Carcinomas[Title/Abstract])) OR (pancreas tumor[Title/Abstract])) OR (pancreas tumour[Title/Abstract]))) | 776 |

**2.Cochrane**

| Search number | Query | Results |
| --- | --- | --- |
| #1 | MeSH descriptor: [Diabetes Mellitus] explode all trees | 47254 |
| #2 | (Diabetes Mellitus):ti,ab,kw OR (Type 2 Diabetes):ti,ab,kw OR (T2DM):ti,ab,kw OR (T1DM):ti,ab,kw OR (Type 1 Diabetes):ti,ab,kw | 96228 |
| #3 | (Diabetes):ti,ab,kw AND (diabetic):ti,ab,kw AND (diabets):ti,ab,kw | 10 |
| #4 | #1 or #2 or #3 | 101103 |
| #5 | MeSH descriptor: [Machine Learning] explode all trees | 1082 |
| #6 | (machine learning):ti,ab,kw OR (risk factors):ti,ab,kw OR (predictors):ti,ab,kw OR (biomarkers):ti,ab,kw OR (prediction model):ti,ab,kw | 170255 |
| #7 | (risk model):ti,ab,kw OR (risk score):ti,ab,kw OR (artificial intelligence):ti,ab,kw OR (Transfer Learning):ti,ab,kw OR (Deep learning):ti,ab,kw | 75129 |
| #8 | (Ensemble Learning):ti,ab,kw OR (random forest):ti,ab,kw OR (neural network):ti,ab,kw OR (neural networks):ti,ab,kw OR (CNN):ti,ab,kw | 4617 |
| #9 | (K-Nearest Neighbor):ti,ab,kw OR (Support vector machine):ti,ab,kw OR (SVM):ti,ab,kw OR (Gradient Boosting Machine):ti,ab,kw OR (Nomogram):ti,ab,kw | 2861 |
| #10 | (XGBoost):ti,ab,kw OR (Adaboost):ti,ab,kw OR (LightGBM):ti,ab,kw OR (CatBoost):ti,ab,kw OR (Gradient Boosting):ti,ab,kw | 345 |
| #11 | (Decision tree):ti,ab,kw OR (Regression Trees):ti,ab,kw OR (ResNet):ti,ab,kw OR (AlexNet):ti,ab,kw OR (VGGNet):ti,ab,kw | 1213 |
| #12 | (GoogLeNet):ti,ab,kw OR (Naive Bayesian):ti,ab,kw OR (Multilayer perceptron):ti,ab,kw OR (Bayesian network):ti,ab,kw OR (Radiomics):ti,ab,kw | 1251 |
| #13 | (Radiomic):ti,ab,kw OR (radiomics-based):ti,ab,kw OR (Texture):ti,ab,kw | 2623 |
| #14 | #5 or #6 or #7 or #8 or #9 or #10 or #11 or #12 or #13 | 221702 |
| #15 | MeSH descriptor: [Pancreatic Neoplasms] explode all trees | 2872 |
| #16 | (Pancreatic Neoplasms):ti,ab,kw OR (Pancreatic Neoplasm):ti,ab,kw OR (Pancreas Neoplasms):ti,ab,kw OR (Pancreas Neoplasm):ti,ab,kw OR (Cancer of Pancreas):ti,ab,kw | 6431 |
| #17 | (Pancreas Cancers):ti,ab,kw OR (Pancreas Cancer):ti,ab,kw OR (Pancreatic Cancer):ti,ab,kw OR (Pancreatic Cancers):ti,ab,kw OR (Cancer of the Pancreas):ti,ab,kw | 6845 |
| #18 | (Islet Cell Carcinoma):ti,ab,kw OR (Islet Cell Carcinomas):ti,ab,kw OR (Pancreatic Intraductal Neoplasms):ti,ab,kw OR (Pancreatic Intraductal Neoplasm):ti,ab,kw OR (Pancreatic IPMN):ti,ab,kw | 150 |
| #19 | (Pancreatic IPMNs):ti,ab,kw OR (Pancreatic Ductal Carcinomas):ti,ab,kw OR (Duct-Cell Carcinoma of the Pancreas):ti,ab,kw OR (Duct Cell Carcinoma of the Pancreas):ti,ab,kw OR (Pancreatic Ductal Carcinoma):ti,ab,kw | 578 |
| #20 | (Ductal Carcinoma of the Pancreas):ti,ab,kw OR (Pancreatic Duct Cell Carcinoma):ti,ab,kw OR (Pancreas Duct-Cell Carcinoma):ti,ab,kw OR (Pancreas Duct-Cell Carcinomas):ti,ab,kw OR (pancreas tumor):ti,ab,kw | 2445 |
| #21 | (pancreas tumour):ti,ab,kw | 2282 |
| #22 | #15 or #16 or #17 or #18 or #19 or #20 or #21 | 8165 |
| #23 | #4 and #14 and #22 | 85 |

**3.Embase**

| Search number | Query | Results |
| --- | --- | --- |
| #31 | #8 AND #25 AND #30 | 2065 |
| #30 | #26 OR #29 | 225245 |
| #29 | 'pancreatic neoplasms':ab,ti OR 'pancreatic neoplasm':ab,ti OR 'pancreas neoplasms':ab,ti OR 'pancreas neoplasm':ab,ti OR 'cancer of pancreas':ab,ti OR 'pancreas cancers':ab,ti OR 'pancreas cancer':ab,ti OR 'pancreatic cancer':ab,ti OR 'pancreatic cancers':ab,ti OR 'cancer of the pancreas':ab,ti OR 'islet cell carcinoma':ab,ti OR 'islet cell carcinomas':ab,ti OR 'pancreatic intraductal neoplasms':ab,ti OR 'pancreatic intraductal neoplasm':ab,ti OR 'pancreatic ipmn':ab,ti OR 'pancreatic ipmns':ab,ti OR 'pancreatic ductal carcinomas':ab,ti OR 'duct-cell carcinoma of the pancreas':ab,ti OR 'duct cell carcinoma of the pancreas':ab,ti OR 'pancreatic ductal carcinoma':ab,ti OR 'ductal carcinoma of the pancreas':ab,ti OR 'pancreatic duct cell carcinoma':ab,ti OR 'pancreas duct-cell carcinoma':ab,ti OR 'pancreas duct-cell carcinomas':ab,ti OR 'pancreas tumor':ab,ti OR 'pancreas tumour':ab,ti | 97473 |
| #26 | 'pancreas tumor'/exp | 216351 |
| #25 | #12 OR #24 | 2477066 |
| #24 | 'machine learning':ab,ti OR 'risk factors':ab,ti OR predictors:ab,ti OR biomarkers:ab,ti OR 'prediction model':ab,ti OR 'risk model':ab,ti OR 'risk score':ab,ti OR 'artificial intelligence':ab,ti OR 'transfer learning':ab,ti OR 'deep learning':ab,ti OR 'ensemble learning':ab,ti OR 'random forest':ab,ti OR 'neural network':ab,ti OR 'neural networks':ab,ti OR cnn:ab,ti OR 'k-nearest neighbor':ab,ti OR 'support vector machine':ab,ti OR svm:ab,ti OR 'gradient boosting machine':ab,ti OR nomogram:ab,ti OR xgboost:ab,ti OR adaboost:ab,ti OR lightgbm:ab,ti OR catboost:ab,ti OR 'gradient boosting':ab,ti OR 'decision tree':ab,ti OR 'regression trees':ab,ti OR resnet:ab,ti OR alexnet:ab,ti OR vggnet:ab,ti OR googlenet:ab,ti OR 'naive bayesian':ab,ti OR 'multilayer perceptron':ab,ti OR 'bayesian network':ab,ti OR radiomics:ab,ti OR radiomic:ab,ti OR 'radiomics based':ab,ti OR texture:ab,ti | 2234150 |
| #12 | 'machine learning'/exp | 529646 |
| #8 | #6 OR #7 | 1621337 |
| #7 | 'diabetes mellitus':ab,ti OR 'type 2 diabetes':ab,ti OR t2dm:ab,ti OR t1dm:ab,ti OR 'type 1 diabetes':ab,ti OR diabetes:ab,ti OR diabetic:ab,ti OR diabets:ab,ti | 1292703 |
| #6 | 'diabetes mellitus'/exp | 1405486 |

**4.Web of science**

| Search number | Query | Results |
| --- | --- | --- |
| 5 | Diabetes Mellitus (Topic) OR Type 2 Diabetes (Topic) OR T2DM (Topic) OR T1DM (Topic) OR Type 1 Diabetes (Topic) OR Diabetes (Topic) OR diabetic (Topic) OR diabets (Topic) | 898077 |
| 6 | machine learning (Topic) OR risk factors (Topic) OR predictors (Topic) OR biomarkers (Topic) OR prediction model (Topic) OR risk model (Topic) OR risk score (Topic) OR artificial intelligence (Topic) OR Transfer Learning (Topic) OR Deep learning (Topic) OR Ensemble Learning (Topic) OR random forest (Topic) OR neural network (Topic) OR neural networks (Topic) OR CNN (Topic) OR K-Nearest Neighbor (Topic) OR Support vector machine (Topic) OR SVM (Topic) OR Gradient Boosting Machine (Topic) OR Nomogram (Topic) OR XGBoost (Topic) OR Adaboost (Topic) OR LightGBM (Topic) OR CatBoost (Topic) OR Gradient Boosting (Topic) OR Decision tree (Topic) OR Regression Trees (Topic) OR ResNet (Topic) OR AlexNet (Topic) OR VGGNet (Topic) OR GoogLeNet (Topic) OR Naive Bayesian (Topic) OR Multilayer perceptron (Topic) OR Bayesian network (Topic) OR Radiomics (Topic) OR Radiomic (Topic) OR radiomics-based (Topic) OR Texture (Topic) | 5778570 |
| 7 | Pancreatic Neoplasms (Topic) OR Pancreatic Neoplasm (Topic) OR Pancreas Neoplasms (Topic) OR Pancreas Neoplasm (Topic) OR Cancer of Pancreas (Topic) OR Pancreas Cancers (Topic) OR Pancreas Cancer (Topic) OR Pancreatic Cancer (Topic) OR Pancreatic Cancers (Topic) OR Cancer of the Pancreas (Topic) OR Islet Cell Carcinoma (Topic) OR Islet Cell Carcinomas (Topic) OR Pancreatic Intraductal Neoplasms (Topic) OR Pancreatic Intraductal Neoplasm (Topic) OR Pancreatic IPMN (Topic) OR Pancreatic IPMNs (Topic) OR Pancreatic Ductal Carcinomas (Topic) OR Duct-Cell Carcinoma of the Pancreas (Topic) OR Duct Cell Carcinoma of the Pancreas (Topic) OR Pancreatic Ductal Carcinoma (Topic) OR Ductal Carcinoma of the Pancreas (Topic) OR Pancreatic Duct Cell Carcinoma (Topic) OR Pancreas Duct-Cell Carcinoma (Topic) OR Pancreas Duct-Cell Carcinomas (Topic) OR pancreas tumor (Topic) OR pancreas tumour (Topic) | 135593 |
| 8 | #7 AND #6 AND #5 | 2318 |
